# Effects of Differing Monomer Compositions on Properties of P(3HB-*co*-4HB) Synthesized by *Aneurinibacillus* sp. H1 for Various Applications

**DOI:** 10.3390/polym14102007

**Published:** 2022-05-13

**Authors:** Aneta Pospisilova, Juraj Vodicka, Monika Trudicova, Zuzana Juglova, Jiri Smilek, Premysl Mencik, Jiri Masilko, Eva Slaninova, Veronika Melcova, Michal Kalina, Stanislav Obruca, Petr Sedlacek

**Affiliations:** Faculty of Chemistry, Brno University of Technology, Purkynova 118, 612 00 Brno, Czech Republic; xcpospisilovaan@fch.vut.cz (A.P.); xcvodickaj@vutbr.cz (J.V.); xctrudicova@fch.vut.cz (M.T.); zuzana.juglova@vut.cz (Z.J.); smilek@fch.vut.cz (J.S.); mencik@fch.vut.cz (P.M.); masilko@fch.vut.cz (J.M.); xcslaninovae@fch.vut.cz (E.S.); xcmelcova@fch.vut.cz (V.M.); kalina-m@fch.vut.cz (M.K.); obruca@fch.vut.cz (S.O.)

**Keywords:** polyhydroxyalkanoates (PHA), P(3HB-*co*-4HB), *Aneurinibacillus* sp. H1, mechanical properties, surface morphology, crystallinity, active ingredients release

## Abstract

Films prepared from poly(3-hydroxybutyrate-*co*-4-hydroxybutyrate) copolymers produced by *Aneurinibacillus* sp. H1 using an automatic film applicator were homogeneous and had a defined thickness, which allowed a detailed study of physicochemical properties. Their properties were compared with those of a poly (3-hydroxybutyrate) homopolymer film prepared by the same procedure, which proved to be significantly more crystalline by DSC and XRD. Structural differences between samples had a major impact on their properties. With increasing 4-hydroxybutyrate content, the ductility and release rate of the model hydrophilic active ingredient increased significantly. Other observed properties, such as the release of the hydrophobic active substance, the contact angle with water and ethylene glycol, or the surface morphology and roughness, were also affected by the composition. The identified properties predetermine these copolymers for wide use in areas such as biomedicine or smart biodegradable packaging for food or cosmetics. The big advantage is the possibility of fine-tuning properties simply by changing the fermentation conditions.

## 1. Introduction

In the course of the last two centuries, plastics have become an inseparable part of human life. The robustness of these materials ensures wide-scale applicability as long-life products. The great variety of its mechanical properties also favours its use in consumer products, such as packages or coatings. However, environmental aspects have played an influential role against petrochemical plastics within the last decades. Therefore, a generation of biodegradable polymers has become a very promising substitute for commercial plastics, especially in the consumption segment. Amongst these polymers, polyhydroxyalkanoates (PHA) attract great attention due to their biocompatibility and full biodegradability [1].

PHAs are a class of polymers that cover a wide range of polymers, including homopolymers and copolymers composed of a large variety of monomers. Numerous monomeric compositions provide great variability in physical and mechanical properties, facilitating several applicability directions. Until now, more than 150 different monomers of PHA have been described; however, only a few of them are relevant to large-scale applications due to production availability and the final impact on the final product properties [2]. The properties of PHA materials are also reflected in the ratio of the monomers in the polymer chain. Hence, material properties could be tailored to specific requirements by managing the polymer composition. However, adjusting the monomer ratio of copolymers is possible, which is quite unique for the biotechnological production of PHA. Fortunately, a thermophilic bacterial strain, *Aneurinibacillus* sp. H1 has appeared as this kind of optimal producent if cultivation conditions are controlled [3]. *Aneurinibacillus* is a remarkable producent of poly(3-hydroxybutyrate-*co*-4-hydroxybutyrate) copolymer able to incorporate 4-hydroxybutyrate (4HB) units up to 90 mol.% by utilising 1,4-butanediol as a precursor, while the monomer ratio in the final copolymer is mirrored to carbon sources (precursor) ratio in the medium [3]. In addition, this thermophilic bacterial strain is also able to produce terpolymers consisting of 3-hydroxybutyrate, 4-hydroxybutyrate, and 3-hydroxyvalerate and does not produce toxic endotoxins [3]. These copolymers seem to be suitable polymers for thin-film production due to their favourable material properties for different applications [4,5].

Polymer films represent a large segment of the industry where the annual consumption of these materials in the EU reaches dozens of megatons. Films are used in packaging, such as insulation or protective layers for sensitive surfaces [6]. The vast majority are made of conventional polymers, especially polyethylene and polypropylene. However, in some segments, there is a demand for biodegradable and biobased alternatives in which PHAs could be applied. Materials of various chemical, optical, and mechanical properties are used [7]. Of the mechanical ones, tear strength, elongation at break, modulus of elasticity, impact strength, tear resistance, and tear propagation are the most important. However, the properties of films also largely depend on additives, surface modifications, and manufacturing methods [8]. Films are usually made by thermoplastic film-forming or solution casting, where the latter option is advantageous for thermally unstable or very high-melting materials [8]. Of the conventional plastics, this applies to cellulose acetate, polyimide, and polycarbonate [9]. The advantage of solution casting is also the possibility of adding temperature-sensitive additives directly during film production. Such additives may improve the physical properties of the film or may be added as active ingredients for sustained release.

Within this paper, we introduce two different P(3HB-*co*-4HB) copolymer films produced by the employment of *Aneurinibacillus* sp. H1 in targeted cultivations and formed by a multi-layer solution casting technique. This production represents a model manufacture of such materials, aiming at an exploration of basic applicability options. As a reference, a homopolymer of 3-hydroxybutyrate was produced by the well-described bacterium *Cupriavidus necator* and further investigated to evaluate the advantages of 4HB incorporation in the polymer chain. Besides the basic physical and mechanical properties, the active ingredient release kinetics is also shown on rhodamine 6G and Nile red. The dyes pose as low molecular solutes, representing active ingredients in medicine or active packaging. The outputs of this research should serve as background for further particular research with respect to specific applications.

## 2. Materials and Methods

The polymer materials used for this study correspond to material used for degradation tests published previously [8]. The materials production was provided by the same procedures. We also introduce some properties relevant to this study, which were previously presented in stated paper, especially the film thickness (determined by mechanical profilometry) and average molecular weight (determined by size exclusion chromatography).

### 2.1. PHA Production

Preparation of the PHAs and polymer films was previously described in [10]. For the purpose of PHAs production, two bacterial strains were employed—*Cupriavidus necator* H16 for P(3HB) biosynthesis and *Aneurinibacillus* sp. H1 for copolymers of 3-hydroxybutyrate and 4-hydroxybutyrate production. To prepare the inoculum, complex media nutrient broth (10 g/L beef extract, 10 g/L peptone, 5 g/L NaCl) were used for bacterial growth. Media were incubated at 30 °C and 45 °C for *Cupriavidus* and *Aneurinibacillus*, respectively, while shaking at 180 rpm for 24 h. Afterwards, mineral salt medium (MSM) was used for PHA production. The inoculum ratio was 10 vol.%. MSM for *C. necator* consisted of the following: KH_2_PO_4_, 1.02 g/L; (NH_4_)_2_SO_4_, 1.0 g/L; Na_2_HPO_4_·12 H_2_O, 11.1 g/L, MgSO_4_·7 H_2_O, 0.2 g/L; trace element solution (TES, composition see Table S1, Supplementary Materials), 1 mL/L; as a carbon source, 20 g/L of fructose has been used. As the MSM for *Aneurinibacillus*, the following components were used: Na_2_HPO_4_·12 H_2_O, 9.0 g/L; KH_2_PO_4_, 1.5 g/L; MgSO_4_·7 H_2_O, 0.2 g/L; NH_4_NO_3_, 1.0 g/L; CaCl_2_·2 H_2_O, 0.02 g/L; FE^III^NH_4_citrate, 0.0012 g/L; Tryptone, 0.5 g/L and microelement solution (MES, composition see Table S2, Supplementary Materials), 1 mL/L. To produce P(3HB-*co*-4HB) copolymer, 1,4-butanediol and glycerol in the following two different ratios were used: 4 g/L of 1,4-BD and 6 g/L of glycerol for lower 4HB-content polymer production, 4 g/L of 1,4-BD and 2 g/L of glycerol for polymer richer in 4HB. Productions were performed in 100 mL Erlenmeyer flasks (250 mL) incubated at 30 °C (*Cupriavidus necator*) and 45 °C (*Aneurinibacillus*) for 72 h under shaking of 180 rpm. Grown media were centrifuged (4800× *g*, 10 min) in order to collect bacterial cells, the biomass was dried at 70 °C in Petri dishes. Acquired biomasses were determined gravimetrically as the cell dry mass (CDM). The PHA content and monomeric composition were determined by gas chromatography as described by Obruca et al.–see Ref. [11]. To obtain the polymers, chloroform extractions were carried out at the SOXTHERM^®^ automatic extraction system (C. Gerhardt Analytical systems, Königswinter, Germany).

### 2.2. Films Production

Preparation of the PHAs and polymer films was previously described in [10]. Briefly, extracted polymeric materials were dissolved in chloroform to 5% *w/w* concentration. Viscous solutions were filtered through 5 µm-nylon filters. To generate films, an automatic film applicator (QTC Sheen, Capelle aan den IJssel, Netherlands) was employed. Each film was prepared by application of two layers of solution (120 µm thickness). After solvent evaporation, film thickness was determined by mechanical profilometry (Bruker, Billerica, MA, USA).

### 2.3. Size Exclusion Chromatography

The method for the average molecular weight determination was described previously [10]. Samples of films were dissolved in HPLC-grade chloroform at a concentration of 1.5 mg/mL. The solutions were filtered through 0.45 µm nylon filters and injected (100 µL) into the size exclusion chromatography system (Infinity 1260 system with PLgel Mixed-C column, Agilent, Santa Clara, CA, USA) coupled with the multi-angle light scattering detector (Dawn Heleos II, Wyatt Technology, Santa Barbara, CA, USA) and differential refractometer (Optilab T-rEX, Wyatt Technology, Santa Barbara, CA, USA). The mobile phase (HPLC-grade chloroform) was pre-filtered through 0.02 µm membrane filters. The flow rate was adjusted to 0.6 mL/min. The raw data were processed in ASTRA software (version 7.3.2, Wyatt Technology, Santa Barbara, CA, USA), the weight-average molecular weight (*M_w_*) was calculated by using Zimm equation.

### 2.4. Atomic Force Microscopy

Surface morphology of P3HB and copolymers P3HB-co-4HB was determined by QI mode (quantitative imaging mode) using JPK Nanowizard 4 AFM (Bruker, Billerica, MA, USA). Rectangular geometry TESPA-V2 (radius 7 nm, resonance frequency 312 kHz, spring constant 33 N/m) was used for the surface imaging of films. Spring constant as well as resonance frequency were experimentally determined during calibration (free oscillation of the probe far enough from the sample surface in the air). P3HB or appropriate copolymers were placed on microscope slide (dried before measurement) and measured in tapping mode under air condition. The following parameters were chosen: setpoint–2.7 nN, Z-speed–50 mm/s, scan size–50 mm, resolution–512 × 512. Images of surface topography were subsequently processed in JPK DP Data Processing software with the following steps: line levelling–linear; smooth QI images–no, interpolation–yes. The surfaces of P3HB and copolymers with 4HB were imagined in pixel difference 3D mode. No further image adjustments were carried out.

### 2.5. Contact Angle Measurements

Sessile drop test was performed on See System E/S (Advex Instruments, Brno, Czech Republic). In total, 5 µL drop of the respective solvent was placed on a polymer film surface using a microsyringe. Contact angles were calculated automatically by the implemented software, See System 7.0 (Advex Instruments, Brno, Czech Republic). Each measurement was repeated seven times.

### 2.6. Tensile Tests

The elongation at break, Young’s modulus, and the tensile strength were determined using the universal testing machine Zwick Z 010 (ZwickRoell GmbH & Co., Ulm, Germany) at ambient conditions. Average values of at least seven measurements were reported. Measurements were carried out using the load indicator with a maximal tensile force of 10 N and with pneumatic grips. Pre-load was 0.1 N and the deformation rate was 5 mm/min. The 5A double-paddle testing samples were cut from the films and heated to 110 °C for 1 h in order to remove residual solvent and enhance crystallisation. Thickness of each specimen was measured before the tensile test.

### 2.7. X-ray Diffractometry (XRD)

XRD patterns were collected using the X-ray diffraction analyzer EMPYREAN (PANalytical, Malvern, UK) in a central focusing arrangement with Bragg-Brentano parafocusing optics using CuKα radiation (range: 5–90° second, step: 0.013° second, voltage: 40 kV, current 30 mA), ADS: 10 mm, time per step: 96 s, without monochromator.

### 2.8. Active Ingredients Release Kinetics

Active ingredients release kinetics was modelled with two different dyes–hydrophilic Rhodamine 6G and hydrophobic Nile red. As a polar environment for Rhodamine release, distilled water was selected. For hydrophobic eluate release, water solution of polyoxyethylene (20) oleyl ether (Brij^®^O20, Sigma Aldrich, St. Louis, MO, USA) was used as the solubilizing agent. Dye was added to polymer solution to consequential concentration of 1 g/L. Subsequently, films were prepared as described above. Pieces of 2.6 × 2.6 cm-coloured films were incubated in solvent for 48 h. Amount of released dye was determined spectrometrically at 524 nm for Rhodamine and 561 nm for Nile red detection.

## 3. Results and Discussion

### 3.1. PHA Films Production

The PHA productivity of *Aneurinibacillus* sp. H1 is generally lower than that of *C. necator*. Compared to *C. necator*, *Aneurinibacillus* sp. H1 reaches lower cell dry mass/biomass yields, and the obtained biomass is quite poor in PHA content as compared to *C. necator* (see Table 1). Furthermore, produced copolymers dispose of a significantly lower average molecular weight than homopolymers, which is given by the difference in PHA synthase types of the respective producents and their general biological dissimilarity (as proposed in [12]), and possible polymerization termination by glycerol [13,14]. On the other hand, manipulating the monomer composition is expected to broaden the range of accessible physical and material properties (crystallinity, ultimate tensile strength), resulting in higher applicability. In addition, thermophilic strains (or generally extremophiles) offer numerous advantages for biotechnological productions if employed, such as lower sterility requirements and fewer cooling needs [15], which partially offset lower process yields, although the production cost is also affected by lower PHA content in the biomass. Hereby, we present basal differences in physical properties in order to illustrate the motivation of P(3HB-*co*-4HB) production by *Aneurinibacillus* sp. H1, since we are able to set the physical properties of the product by a simple choice of cultivation strategy. Accordingly, we would like to point out the first two columns of Table 1 as the key results presented here. Therefore, not all of the stated aspects are accountable or projectable in the final production costs and the stimulus of P(3HB-*co*-4HB) copolymer production by *Aneurinibacillus* sp. H1 should definitely follow the requirements on final product properties (purposes) presented in this paper.

### 3.2. Surface Characteristics

**Figure 1:**
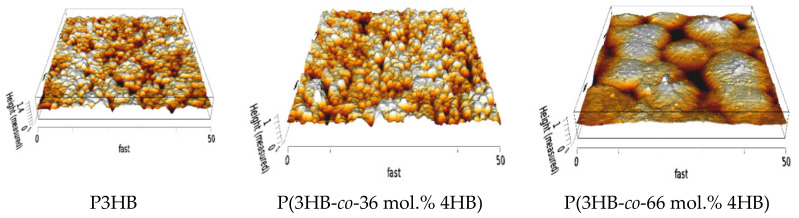
AFM surface morphology of prepared films illustrating the differences in the surface roughnesses of P3HB, P(3HB-*co*-36 mol.% 4HB) and P(3HB-*co*-66 mol.% 4HB).

The films were prepared using a film applicator with a controlled thickness of the solution layer. This enabled us to prepare uniform and well-defined films with good control over the film thickness (Table 2). Certain dissimilarities were observed by AFM, where different copolymers had slightly different surface morphologies (see Figure 1). This could be caused by differences in molecular weight, crystallinity, and rate of crystal formation.

### 3.3. Contact Angle

**Figure 2:**
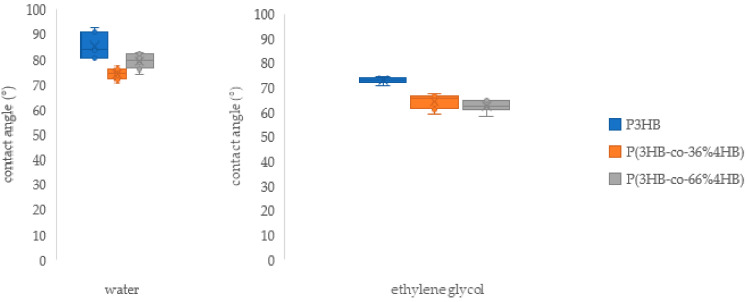
Contact angles of prepared films suggesting differences in the surface wettability of the homopolymer and the copolymers films.

The contact angle is a basic indicator of the wettability and a good measure of the hydrophilicity of the material. It is affected by surface free energy, liquid surface tension, and roughness [16]. The interaction of the material with solvents plays a major role in a number of technological steps involved in film production and use, such as cleaning, gluing, printing, and dyeing [17]. The contact angles of the tested PHA are presented in Figure 2.

**Figure 3:**
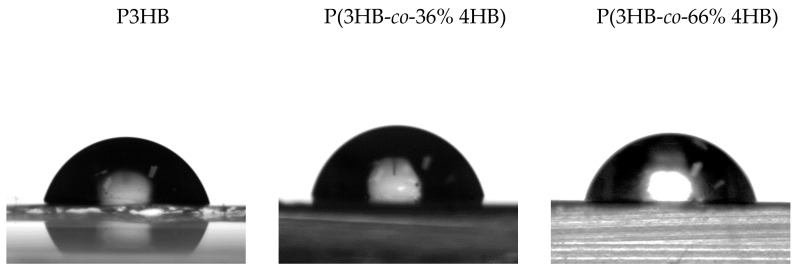
Images from water contact angle measurements representing high hydrophobicity of all tested films–P3HB, P(3HB-*co*-36 mol.% 4HB) and P(3HB-*co*-66 mol.% 4HB).

Both P3HB and P(3HB-*co*-4HB) copolymers have been confirmed to be relatively hydrophobic materials that have a rather high contact angle when in contact with polar liquids (water and ethylene glycol, see Figure 3). The results are comparable with data obtained by other authors on similar PHAs [18,19,20,21]. P3HB and P(3HB-*co*-4HB)s are similar to synthetic polymers, which are commonly used in film technology [22], and significantly differ from other natural polymers (polysaccharides, proteins, nucleic acids, etc.) that are highly hydrophilic. This can make the PHAs a suitable alternative to synthetic plastics in applications where surface hydrophobicity plays an important role. An example could be the packaging of hydrophilic foodstuffs—hydrophobic packaging is generally more advantageous than hydrophilic because strong adhesion of the content to packaging may lead to impaired product acceptability and increased waste [16].

### 3.4. Tensile Test

**Figure 4:**
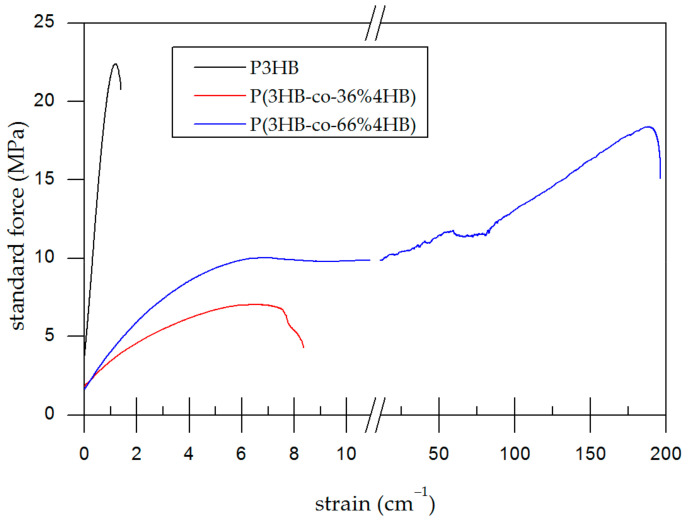
Typical stress−strain curves demonstrating differences in ducilities of the tested homopolymer (P3HB) and the P(3HB-*co*-4HB) copolymers films.

Tensile testing (Table 3) showed the dependence of mechanical properties on the content of 4HB in the copolymer, as apparent from Figure 4. With increasing 4HB content, the films were more ductile, which can be attributed to substantially lower crystallinity. We have seen that the samples change significantly over time. Immediately after preparation, all samples were much less stiff than after several days of storage at room temperature (data not shown). The properties did not seem to stabilize within two weeks of storage. From the perspective of film applications, it is important to estimate the properties of the polymer in the weeks to months after preparation. Therefore, to ensure complete crystallisation and removal of residual solvent, the samples were pre-treated by heating at 110 °C for 1 h. We assume that this may explain the difference between our data and the results of other authors, who usually observed much higher ductility. The authors pre-treated samples by prolonged storage at room temperature [23,24,25,26] or did not specify the pre-treatment procedure [27,28]. Another reason may be the different monomer sequence of the copolymers, different molecular weight, or different parameters of the measurement. Anyway, the copolymers are more ductile than pure P3HB, retain their ductility even after a long time, and the degree of ductility is adjustable by the 4HB content, which depends on the fermentation conditions. This is beneficial for the fine-tuning of properties in applications where the ductility of material and its control is crucial.

### 3.5. X-ray Diffraction Patterns

**Figure 5:**
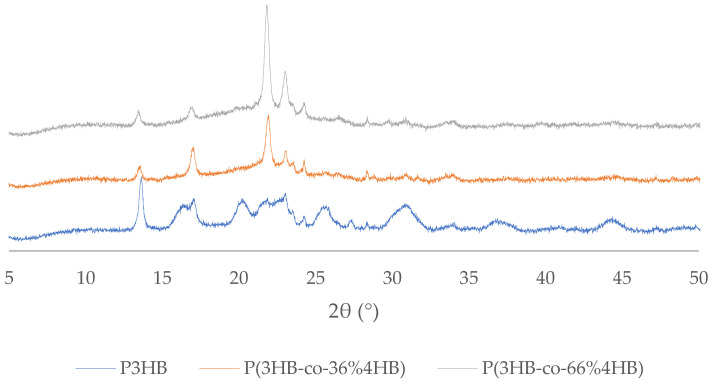
X-ray Diffraction patterns indicating different crystalline morphology of P3HB and the P(3HB-*co*-4HB) copolymers.

The obtained diffraction patterns (see Figure 5) revealed that all materials were semicrystalline with characteristic diffractions for crystalline phases and a broad background band attributable to the amorphous fraction. The ratio between P3HB and P4HB crystallites gradually shifted with changing copolymer composition, confirming the copolymer composition found by the GC-FID method. The diffraction patterns correspond well with previously described data [12] obtained on materials produced by the same microorganism as in this work. Other authors (see Ref. [26]) achieved slightly different patterns for the copolymers, with a substantially lower ratio of P3HB crystallites in the P(3HB-*co*-66% 4HB) copolymer. It reveals that not only copolymer composition determines polymer properties–other factors, such as distribution of the monomers in the polymer chains (random or block) or molecular weight, may also affect the properties.

### 3.6. Active Ingredients Release Kinetics

Biopolymers recently took a position among potential reservoirs for active ingredients’ release. These systems find applications in packaging technologies, such as active or intelligent packaging of foods, cosmetics, pharmaceuticals, or other consumer products. Concurrently, these polymers as active ingredient reservoirs are potential matrices for the ingredient release in various environments (agricultural, synthetic, physiologic, etc.). Several requirements are put on matrices for active ingredient release. In general, the matrix should be able to hold high amounts of the ingredient and optimally release the whole amount of the ingredient in the defined time period (to ensure no ingredient wasting). The rate of ingredient release strongly depends on the matrix properties. Thus, we assume that the monomer composition of tested PHAs will drive the final release behaviour.

**Figure 6:**
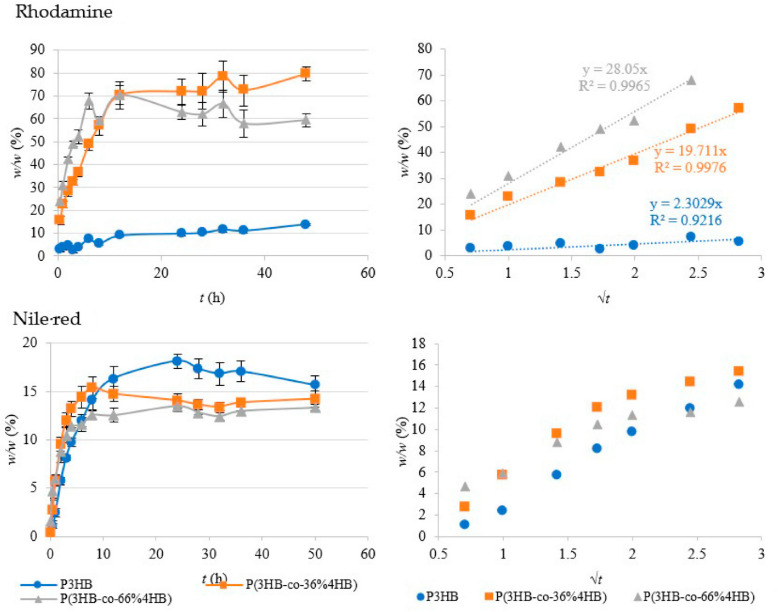
Results from active ingredients release tests highlighting the differences in rhodamine and Nile red release from the homopolymer P3HB and the P(3HB-*co*-4HB) copolymers.

Rhodamine’s structure combines aromatic rings and positive charge, which is analogous to common pharmaceuticals, for instance, antibiotics or anaesthetics [29,30]. The results of rhodamine release (see Figure 6) show a clear difference in both the course of elution and the overall amount of dye released for homopolymers and copolymers. Apparently, the crystalline morphology of both copolymers is much more favourable for hydrophilic low-molecular substances release than homopolymer structure since facilitates fast elution to the aqueous environment. The 4HB content in the polymer matrix obviously enhances the usability of the hydrophilic active ingredient and ensures the release of significantly higher amounts of the ingredient. Moreover, the rate of release is predictable by shifting the 3HB:4HB ratio. On the other hand, P3HB shows better retention of the solute and a stable (slow) release of ingredients. The linear regression (dependence of *w*/*w* on *√t*, Figure 6) confirmed that diffusion is the rate-limiting step. The diffusion is apparently driven by different diffusion coefficients D for individual matrices, as observed on different curve slopes. These are the aspects to consider individually for intended applications. The release from P(3HB-*co*-66% 4HB) is more rapid within the first 6 h compared to P(3HB-*co*-36% 4HB), whilst the other dominates in the following “plateau” phase. Though, it is not possible to formulate strict conclusions about the phenomena in the plateau phase since the deviations are quite high in this phase.

Nile red as hydrophobic active ingredient represents model of typical pharmaceutical ingredient (e.g., analgesic), where low water solubility is very common. Compared to rhodamine release, Nile red release did not reach high values of the final amount of solute release (Figure 6). Even though this conclusion is justified (due to low dye polarity), the final amount of the released Nile red is surprising since it would be expected to appear up to 10%. The final concentration of approximately 2.9 µM and 2.3 µM of released Nile red from the homopolymer and copolymers respectively approaches the maximal solubility in water–approx. In total, 3 µM (according to Ref. [31]). Probably, the matrix–water systems achieved equilibrium after the solvent saturation, so this resulted in limited the elution. Obviously, not even the presence of the high-molecular wetting agent contributed to the hydrophobic dye release.

The rate of release of Nile red within the exponential phase is basically the same for copolymers and homopolymer as shown on the Figure 6. Only very slight differences are observable in the time dependence, where homopolymer reports very short “lag time” compared to copolymers, and former incline to plateau phase of P(3HB-co-66% 4HB). For this reason, the regressions (square root dependence) are not displayed. Though, the differences between homopolymer and copolymer are almost insignificant compared to Rhodamine release. The diffusion rate is generally influenced by diffusion coefficient D and the concentration gradient as the main stimuli. Though, the homopolymer consisting of higher number of crystalline regions potentially retaining the hydrophobic dye might represents matrix reducing D, the high-crystalline regions are capable aggregate the dye during matrix formation (load) so cumulating local over-gradients possibly compensating diffusivity deficiency.

## 4. Conclusions

Within this study, we have applied the previously described model of P(3HB-*co*-4HB) copolymer production by the thermophilic bacterium *Aneurinibacillus* sp. H1 (see Refs. [3,12]) to adjust selected product properties via different cultivation strategies. Compared to P3HB production by *C. necator*, the productivity of the copolymer synthesis is considerably lower. On the contrary, manufactured copolymers showed a significant advance in mechanical properties as they incorporated a higher number of 4HB units. Elongation at break of P(3HB-*co*-66% 4HB) reached about a hundred times higher values than P3HB, which predetermines this copolymer for the production of high-value packaging technology (active, smart, or bioresorbed coatings), for instance for foodstuffs, cosmetics, and consumer products, where high flexibility of foils is required. Further, the surface of copolymers appears slightly less hydrophobic as the contact angles with water and ethylene glycol are gently lower compared to homopolymers. This ensures higher reactivity of the surface on the interface with water environments, possibly broadening the applicability of copolymers in biomedicine (degradable soft tissue implants and scaffolds, surgical sutures, repair patches, etc.). Suitability for in vivo applicability was also confirmed by active ingredients release testing, where copolymers showed rapid ingredient release when releasing both hydrophilic and hydrophobic low-molecular ingredients. In addition, the release of hydrophilic rhodamine from copolymer matrices might prevent wasting of the active ingredient since the matrices allow high effective leaching of the dye. The modelling of copolymer content as a reservoir could be beneficially used for various kinetics of in vivo drug release or active packaging. In our previous study (see Ref. [10]), we reported the high biodegradability of tested copolymers in simulated physiological environments and found no cytotoxicity of the degradation products. A combination of these outputs shows great usability of tested P(3HB-*co*-4HB) copolymers in biomedicine and foodstuff or cosmetics packaging.

## Figures and Tables

**Table 1:** Results of polymers production–also in [10].

Sample	Substrate	CDM (g/L)	PHA (% per CDM)	*M_w_* (kDa)	Crystallinity ^+^ (%)
P3HB	fructose	9.06 ± 0.24	76.82 ± 12.44	481.26 ± 11.62	59
P(3HB-*co*-36% 4HB) *	1,4-BD:GLY (2:3)	1.28 ± 0.53	29.46 ± 3.67	127.14 ± 1.73	36
P(3HB-*co*-66% 4HB) *	1,4-BD:GLY (2:1)	1.82 ± 0.18	32.09 ± 3.16	174.13 ± 4.27	43

1,4-BD = 1,4-butanediol; GLY = glycerol; CDM = cell dry mass; *M_w_* = weight average molecular weight; * Copolymer composition determined by GC-FID; ^+^ Counted from melting enthalpies (146 J/g and 110 J/g for 100% crystalline P3HB and P4HB, respectively [12]).

**Table 2:** Data from mechanic profilometry.

Sample	Film Thickness (µm)	Roughness Ra (µm)
P3HB	10.96 ± 1.11	0.35
P(3HB-*co*-36% 4HB)	11.12 ± 1.37	0.67
P(3HB-*co*-66% 4HB)	11.09 ± 0.57	0.20

**Table 3:** Results from the tensile tests.

Sample	*E_t_* (MPa)	*σ_m_* (MPa)	*ε_m_* (%)
P3HB	2000 ± 167	20.4 ± 1.82	1.3 ± 0.12
P(3HB-*co*-36% 4HB)	161 ± 14.7	6.72 ± 0.667	5.3 ± 0.91
P(3HB-*co*-66% 4HB)	210 ± 35.8	13.8 ± 3.63	180 ± 52.0

## Data Availability

The data that support the findings of this study are available from the corresponding author, P.S., upon reasonable request.

## Supplementary Materials

The following supporting information can be downloaded at: https://www.mdpi.com/article/10.3390/polym14102007/s1, Table S1: Composition of microelement solution (MES); Table S2: Composition of trace element solution (TES).
