# Supplementary material for: Effects of Differing Monomer Compositions on Properties of P(3HB-co-4HB) Synthesized by Aneurinibacillus sp. H1 for Various Applications
Source: Polymers (Basel). 2022 May 13;14(10):2007. doi: 10.3390/polym14102007 (PMC9146627; doi:10.3390/polym14102007)
Supplement: Supplementary file 1 [file polymers-14-02007-s001.zip › polymers-1707374-supplementary.pdf]

polymers

Supplementary material

Effects of Differing Monomer Compositions on Properties of P(3HB-*co*-4HB) Synthesized by *Aneurinibacillus* sp. H1 for Various Applications

Aneta Pospisilova, Juraj Vodicka, Monika Trudicova, Zuzana Juglova, Jiri Smilek, Premysl Mencik, Jiri Masilko, Eva Slaninova, Veronika Melcova, Michal Kalina, Stanislav Obruca and Petr Sedlacek *

Faculty of Chemistry, Brno University of Technology, Purkynova 118, 612 00 Brno, Czech Republic; xcpospisilovaan@fch.vut.cz (A.P.); xcvodickaj@vutbr.cz (J.V.); xctrudicova@fch.vut.cz (M.T.); zuzana.juglova@vut.cz (Z.J.); smilek@fch.vut.cz (J.S.); mencik@fch.vut.cz (P.M.); masilko@fch.vut.cz (J.M.); xcslaninovae@fch.vut.cz (E.S.); xcmelcova@fch.vut.cz (V.M.); kalina-m@fch.vut.cz (M.K.); obruca@fch.vut.cz (S.O.)

* Correspondence: sedlacek-p@fch.vut.cz; Tel.: +420-541-149-486

Table S1. Composition of microelement solution (MES).

Component	Amount [g/L]
$FeCl_3 \times 6 \times H_2O$	9.7
$CaCl_2 \times 2 \times H_2O$	7.8
$CuSO_4 \times 5 \times H_2O$	0.156
$CoCl_2 \times 6 \times H_2O$	0.119
$NiCl_2 \times 6 \times H_2O$	0.118
$CrCl_2$	0.062
0,1 M HCl	1000 mL

Table S2. Composition of trace element solution (TES).

Component	Amount [g/L]
EDTA	50
$FeCl_3$	8.3
$ZnCl_2$	0.84
$CuCl_2 \times 2 \times H_2O$	0.13
$CoCl_2 \times 6 \times H_2O$	0.1
$MnCl_2 \times 6 \times H_2O$	0.016
$H3BO_3$	0.1
Water	1000 mL
